# Associations of Internet Addiction Severity With Psychopathology, Serious Mental Illness, and Suicidality: Large-Sample Cross-Sectional Study

**DOI:** 10.2196/17560

**Published:** 2020-08-11

**Authors:** Wanjun Guo, Yujie Tao, Xiaojing Li, Xia Lin, Yajing Meng, Xia Yang, Huiyao Wang, Yamin Zhang, Wanjie Tang, Qiang Wang, Wei Deng, Liansheng Zhao, Xiaohong Ma, Mingli Li, Ting Chen, Jiajun Xu, Jing Li, Wei Hao, Sing Lee, Jeremy W Coid, Andrew J Greenshaw, Tao Li

**Affiliations:** 1 Mental Health Center and Psychiatric Laboratory State Key Laboratory of Biotherapy West China Hospital of Sichuan University Chengdu, Sichuan China; 2 Institute of Emergency Management and Post-Disaster Reconstruction Sichuan University Chengdu China; 3 North Sichuan Medical College (NSMC) Affiliated Hospital of North Sichuan Medical College Nanchong City China; 4 Centre for Educational and Health Psychology Sichuan University Chengdu China; 5 Mental Health Institute The Second Xiangya Hospital Central South University Hunan, Changsha China; 6 Department of Psychiatry The Chinese University of Hong Kong Hong Kong Hong Kong; 7 Department of Psychiatry University of Alberta Edmonton, AB Canada

**Keywords:** internet, addiction, psychopathology, suicidality, serious mental illness

## Abstract

**Background:**

Internet addiction has become a major global concern and a burden on mental health. However, there is a lack of consensus on its link to mental health outcomes.

**Objective:**

The aim of this study was to investigate the associations between internet addiction severity and adverse mental health outcomes.

**Methods:**

First-year undergraduates enrolled at Sichuan University during September 2015, 2016, 2017, and 2018 were invited to participate in the current study survey, 85.13% (31,659/37,187) of whom fully responded. Young’s 20-item Internet Addiction Test, Patient Health Questionnaire-15, Patient Health Questionnaire-9, Symptom Checklist 90, Six-Item Kessler Psychological Distress Scale, and Suicidal Behaviors Questionnaire-Revised were used to evaluate internet addiction, four psychopathologies (high somatic symptom severity, clinically significant depression, psychoticism, and paranoia), serious mental illness, and lifetime suicidality.

**Results:**

The prevalence of students with mild, moderate, and severe internet addiction was 37.93% (12,009/31,659), 6.33% (2003/31,659), and 0.20% (63/31,659), respectively. The prevalence rates of high somatic symptom severity, clinically significant depression, psychoticism, paranoid ideation, and serious mental illness were 6.54% (2072/31,659), 4.09% (1294/31,659), 0.51% (160/31,659), 0.52% (165/31,659), and 1.88% (594/31,659), respectively, and the lifetime prevalence rates of suicidal ideation, suicidal plan, and suicidal attempt were 36.31% (11,495/31,659), 5.13% (1624/31,659), and 1.00% (315/31,659), respectively. The prevalence rates and odds ratios (ORs) of the four psychopathologies and their comorbidities, screened serious mental illness, and suicidalities in the group without internet addiction were much lower than the average levels of the surveyed population. Most of these metrics in the group with mild internet addiction were similar to or slightly higher than the average rates; however, these rates sharply increased in the moderate and severe internet addiction groups. Among the four psychopathologies, clinically significant depression was most strongly associated with internet addiction after adjusting for the confounding effects of demographics and other psychopathologies, and its prevalence increased from 1.01% (178/17,584) in the students with no addiction to 4.85% (582/12,009), 24.81% (497/2,003), and 58.73% (37/63) in the students with mild, moderate, and severe internet addiction, respectively. The proportions of those with any of the four psychopathologies increased from 4.05% (713/17,584) to 11.72% (1408/12,009), 36.89% (739/2003), and 68.25% (43/63); those with lifetime suicidal ideation increased from 24.92% (4382/17,584) to 47.56% (5711/12,009), 67.70% (1356/2003), and 73.02% (46/63); those with a suicidal plan increased from 2.59% (456/17,584) to 6.77% (813/12,009), 16.72% (335/2003), and 31.75% (20/63); and those with a suicidal attempt increased from 0.50% (88/17,584) to 1.23% (148/12,009), 3.54% (71/2003), and 12.70% (8/63), respectively.

**Conclusions:**

Moderate and severe internet addiction were strongly associated with a broad group of adverse mental health outcomes, including somatic symptoms that are the core features of many medical illnesses, although clinically significant depression showed the strongest association. This finding supports the illness validity of moderate and severe internet addiction in contrast to mild internet addiction. These results are important for informing health policymakers and service suppliers from the perspective of resolving the overall human health burden in the current era of “Internet Plus” and artificial intelligence.

## Introduction

The internet has conceptually transformed the Earth into a high-dimensional information network village, and human experience has benefited from the unprecedented availability and exchange of information. However, the potential adverse effects of internet exposure on human health have emerged as a major global concern [1].

In June 2017, the English addiction therapist Mandy Saligari warned parents that giving a smartphone to their child may be as damaging as giving them “a gram of cocaine” due to its potential addictive features and association with adverse mental consequences [2]. In September 2017, the French government banned students from using mobile phones in the country’s primary, junior, and middle schools [3]. In China, the world’s largest market for online gaming since 2015 due to its high population size and ubiquitous national internet access [4] (China’s 4G network covered 95% of its administrative villages and 99% of its population by the end of April 2018 [5]), overuse of internet services such as gaming and social media, and the association of overuse with adverse mental consequences among youth has received great public attention. News associated with possible internet addiction–associated adverse events (eg, decline in school grades; self-harm, including suicide and accidental death relevant to internet addiction; and “digital detox” boot camps to treat internet addiction in isolated settings) has dramatically increased in recent years, and some Chinese parents and teachers have depicted internet addiction as “electronic opium” or “electronic heroin” [6,7]. Accordingly, in April 2018, the Ministry of Education of China released an urgent notice concerning prevention of primary and middle school students from indulging in the internet [8].

Since Dr. Kimberly Young published a case report in 1996, internet addiction, sometimes called internet dependence or problematic internet use (PIU), has been increasingly conceptualized as “a kind of psychopathological disorder” [9,10]. Studies have found that internet addiction appears to share a phenomenology akin to that of impulse control/addictive disorders [11]. Some studies have even documented possible biomarkers for this condition [12]. The prevalence of internet addiction is high in some young populations and may have increased in recent years, although this prevalence varies regionally around the world [13]. Further research has suggested that internet addiction may be associated with significant functional and psychological impairment, and may be a contributing factor to issues of interparental conflict, family dissatisfaction, recent stressful events, low reward dependence, and low self-esteem, which all may further increase vulnerability to internet addiction [14,15].

After a wide-ranging debate on whether internet addiction primarily reflects the adverse effects of internet contents (such as gaming and social media as opposed to information seeking) or technologies [16-20], some internet addiction–related types have been identified as official mental disorders in recent revisions of international disease classification and diagnostic instruments. The Diagnostic and Statistical Manual of Mental Disorders (DSM)-5 included internet gaming disorder (IGD) in the appendix as a disorder requiring further study [21]. The International Classification of Diseases (ICD)-11 defined and specified gaming disorder as “predominantly online” or “predominantly offline” [22]. Some researchers have argued that classification of IGD or gaming disorder as predominantly online is premature. This is based on several concerns, including low quality/extent of available research, the fact that current operationalization leans too heavily on substance use and may be locked into a confirmatory approach rather than an exploration of the boundaries of normal vs pathological behavior [23], and a lack of consensus on the link of internet addiction or specific internet addiction patterns (including IGD) with other mental health outcomes [19,24]. Some cross-sectional studies found that internet addiction (or PIU) was significantly associated with poor sleep quality [18], attention-deficit/hyperactivity disorder (ADHD) symptoms [25], anxiety [25], depression [25], and even suicidality [26]. In a longitudinal study, Lau et al [27] found a bidirectional predictive relationship between internet addiction and depression. A higher level of hyperactivity/inattention and self-esteem problems were predictors of IGD, and IGD was a predictor of adolescent emotional distress in the context of further longitudinal work [28]. Some studies suggest that, relative to internet addiction, the association of problematic online gaming might be stronger with hyperactivity/inattention but less strong with other common psychopathologies such as depression [19,20]. However, these association studies suffer from two major limitations: (1) their relatively small sample sizes (from hundreds to approximately 3000 participants) precluded an analysis of the rates and odds ratios (ORs) of linked mental health outcomes based on detailed internet addiction severity rather than dichotomous decisions (ie, either a person has or does not have internet addiction or PIU), and (2) each of these studies investigated only a single or a small number of psychopathologies rather than a series of representative syndromes. This latter limitation has precluded the observation of variance in the association patterns and comorbidity across syndromes.

To overcome these limitations, in the present study, we investigated the prevalence of four representative psychopathologies (high somatic symptom severity, clinically significant depression, psychoticism, and paranoid ideation), which could reflect a broad range of symptoms and different levels of severity of clinical manifestation of mental disorders, as well as serious mental illness and suicidality among groups with different levels of internet addiction severity. The study was conducted using a large Chinese undergraduate sample comprising the largest sample evaluated in internet addiction research to date.

## Methods

### Study Design and Ethics

The present study, which was approved by the Ethics Committee of West China Hospital, Sichuan University (no. 2016-171), is based on the cross-sectional data from the Online Psychosomatic Health Survey (OPHS) system of Sichuan University. Sichuan University is a national comprehensive university located in southwestern China whose undergraduates come from all of the provincial administrative regions of China. Every respondent was informed of the aim of this investigation prior to the formal survey, and internet-based informed consent was obtained from each participant.

### Survey Development

The questions and scales in the OPHS system were designed and selected by a group of researchers from the Mental Health Center, West China Hospital of Sichuan University, and the Centre for Educational and Health Psychology of Sichuan University. This survey was conducted through internet messages with a website link. Students voluntarily logged in with their student ID and were able to quit at any time without penalty. The final survey domains included in the OPHS system were: sociodemographics, childhood adversity, Adolescent Self-Rating Life Events Check List, psychopathologies (including high somatic symptom severity, clinically significant depression, psychoticism, and paranoid ideation), psychological distress, suicidality, and internet usage. In addition to demographics of biological sex and age (since the age for most students that attend college is 18 years old in China, the respondents were categorized into three groups: younger than, equal to, or older than 18 years old), data were collected using the Young 20-item Internet Addiction Test (IAT), Patient Health Questionnaire-15 (PHQ-15), Patient Health Questionnaire-9 (PHQ-9), psychoticism and paranoid ideation subscales of the Symptom Checklist 90 (SCL-90), six-item Kessler psychological distress scale (K6), and Suicidal Behaviors Questionnaire-Revised (SBQ-R) for the specified research aims. The raw data were saved and stored in a manner only accessible by the research administrators.

### Participants and Survey Administration

All undergraduate freshmen enrolled in university during September 2015, September 2016, September 2017, and September 2018 were invited to participate. For inclusion in the study, participants were required to finish the entire self-administered questionnaire on the internet by October 31 of their year of enrollment. Altogether, 37,187 freshmen were invited to participate, 34,140 (91.80%) of whom agreed to participate and logged on to the online survey system. Participants in this study were excluded for the following reasons: failure to complete all of the survey questions, taking less than 10.0 minutes to finish the survey (the median time to finish the survey was 30.6 minutes), did not turn in the questionnaire by November, were not between 15 and 23 years old, or provided obvious dishonest information (eg, selected the same severity option for all questions in a separate scale or questionnaire, or a unique ID was logged in by two or more participants). The final sample comprised 31,659 respondents, yielding an overall effective response rate of 85.13%.

### Measurements

#### Young IAT

Young IAT has been internationally validated to assess internet addiction [29,30]. The IAT consists of 20 questions asking about the extent of an individual’s involvement with the internet, with each question rated on a scale of 1 to 5, in addition to the rating of “not applicable” as 0. Total scores can range from 0 to 100, with the severity of internet addiction categorized as normal (0-30), mild (31-49), moderate (50-79), and severe (80-100) [9]. Cronbach α for this questionnaire in this study was .90.

#### PHQ-15

The PHQ-15, based on the diagnostic criteria of depression from DSM-IV, was used to measure high somatic symptom severity of participants in this study. PHQ-15 is a self-administered, internationally validated, and widely used questionnaire for screening somatization and monitoring somatic symptom severity in clinical practice and research. Its test-retest reliability coefficient was found to be 0.75 in a Chinese population [31,32]. Subjects were asked to rate the severity of 13 somatic symptoms as 0 (“not bothered at all”), 1 (“bothered a little”), or 2 (“bothered a lot”) during the 4 weeks preceding the study. Two additional physical symptoms—feeling tired or having little energy and trouble sleeping—were coded as 0 (“not at all”), 1 (“several days”), or 2 (“more than half the days” or “nearly every day”). The range of the scale is 0 to 30 and we used a conventional cutoff of ≥10 to define high somatic symptom severity. Cronbach α for the questionnaire in this study was .79.

#### PHQ-9

The nine questions of PHQ-9 ask about the participant’s experience in the last 2 weeks, with the possible responses ranging from 0 (“not at all”) to 3 (“nearly every day”) and a score ranging from 0 to 27. A cutoff of ≥10, reported as the optimal cutoff to detect major depressive episodes in the Chinese population, was used to define clinically significant depression in the present study [33]. Cronbach α for the questionnaire in this study was .84.

#### Psychoticism and Paranoid Ideation Subscales of the SCL-90

Subscales of the SCL-90 were used to assess psychoticism and paranoid ideation in this study. High validity and reliability of SCL-90 has been demonstrated both in the Chinese general population and with university students [34,35]. The psychoticism and paranoid ideation subscales comprise 10 and 6 specific symptoms, respectively. Responses fit on a 5-point scale, from 0 (“not at all”) to 4 (“extremely”) for both subscales. Each subscale score was calculated as sum score/item numbers. Mean normative scores (1.50, SD 0.51 for psychoticism and 1.63, SD 0.57 for paranoid ideation based on meta-analysis results of studies on Chinese university students) were used to define psychoticism and paranoid ideation [35], yielding subscale score criteria of >2.01 and >2.20 to identify individuals as having psychoticism and paranoid ideation, respectively. Cronbach α of the subscales of psychoticism and paranoid ideation was .83 and .80, respectively, in this study.

#### K6

The K6 is a self-report 6-item scale that asks respondents to rate how frequently they have felt “nervous,” “hopeless,” “restless or fidgety,” “so depressed that nothing could cheer you up,” “that everything was an effort,” and “worthless” during the past 30 days. The 5-point response options for each question ranged from 0 (none) to 4 (all of the time) [36]. For the Chinese version, reported validity in both the general population and university students was good for rapidly screening serious mental illness, which has been defined as meeting the criteria for one or more DSM-IV/Composite International Diagnostic Interview (CIDI) mental disorders and resulting in serious impairment [36,37]. The optimal cutoff of 12/13, identified by these studies, was the criterion for serious mental illness in the present study. Cronbach α for the questionnaire in this study was .86.

#### SBQ-R

In this study, the first SBQ-R question was used to survey lifetime suicidality in participants. This self-report questionnaire consists of 4 questions used to identify suicide risk and has been validated for use with university students. Both single SBQ-R Item 1 and SBQ-R total scores have been recommended for use in clinical and nonclinical settings [38]. Question 1 explores whether the respondent has ever entertained risky thoughts of suicide or attempted to kill himself/herself in his/her lifetime. Response options include “1 (never),” “2 (It was just a brief passing thought),” “3a (I have had a plan at least once to kill myself but did not try to do it),” “3b (I have had a plan at least once to kill myself and really wanted to die),” “4a (I have attempted to kill myself but did not want to die),” and “4b (I have attempted to kill myself, and really hoped to die).” The present study defined scores of ≥2, ≥3, and 4 as suicidal ideation, suicidal plan, and suicidal attempt, respectively.

### Statistical Analysis

Data analyses were conducted using SPSS 22.0 software. The mean (95% CI) and median age, number of respondents in each demographic and internet addiction severity group, and prevalence rates (95% CIs) across levels of internet addiction severity, psychopathology, serious mental illness, and suicidality were calculated. The Chi-square test was used to compare prevalence rates among demographic or internet addiction severity groups, and the adjusted ORs and their 95% CIs of other mental health outcomes among the internet addiction severity groups were estimated and compared using binary logistic regression, adjusting for confounding effects of demographics (ie, age, sex, and enrollment year) and the interactions among psychopathologies. A two-tailed alpha level of .05 was used to evaluate statistical significance.

## Results

### Demographics

The 31,659 respondents included 16,109 (50.88%) men and 15,550 (49.12%) women. The groups aged 15-17 years, 18 years, and 19-23 years included 4821 (15.23%), 19,034 (60.12%), and 7804 (24.65%) respondents, respectively.

### Prevalence of Different Severities of Internet Addiction

Overall, the rates of those without internet addiction and those with mild, moderate, and severe internet addiction were 55.5%, 37.9%, 6.3%, and 0.2%, respectively. Internet addiction severity rates differed between sexes, among the age groups, and among the year-of-survey groups. The rate of moderate internet addiction was higher in women than in men. The oldest group (aged 19-23 years) included more respondents without internet addiction and fewer individuals with mild internet addiction than the other two age groups. Among the year-of-survey groups, the rates of all levels of internet addiction severity increased with recency (Table 1).

**Table 1:** Prevalence (%) and severities of internet addiction in the surveyed population.

Subgroup		Mild internet addiction					Moderate internet addiction					Severe internet addiction					χ^2^			df			*P* value
			n (%)		95% CI		n (%)		95% CI		n (%)			95% CI									
Whole sample (N=31,659)			12,009 (37.93)		37.40-38.47		2003 (6.33)		6.06-6.60		63 (0.20)			0.15-0.25									
**Sex**															36.02			3			<.001		
	Male (n= 16,109)		5992 (37.20)		36.45-37.94		920 (5.71)		5.35-6.07		29 (0.18)			0.11-0.25									
	Female (n= 15,550)		6017 (38.70)		37.93-39.46		1083 (6.96)		6.56-7.36		34 (0.22)			0.15-0.29									
**Age (years)**															27.51			6			<.001		
	15-17 (n= 4821)		1877 (38.93)		37.56-40.31		342 (7.10)		6.37-7.82		5 (0.10)			0.01-0.19									
	18 (n= 19,034)		7309 (38.40)		37.71-39.09		1198 (6.29)		5.95-6.64		39 (0.20)			0.14-0.27									
	19-23 (n= 7804)		2823 (36.17)		35.11-37.24		463 (5.61)		5.09-6.12		19 (0.24)			0.13-0.35									
**Year of survey**															272.32			9			<.001		
	2015 (n= 8723)		3167 (36.31)		35.30-37.32		330 (3.78)		3.38-4.18		6 (0.07)			0.01-0.12									
	2016 (n= 6501)		2318 (35.66)		34.49-36.82		435 (6.69)		6.08-7.30		16 (0.25)			0.13-0.37									
	2017 (n= 8644)		3291 (38.07)		37.05-39.10		593 (6.86)		6.33-7.39		18 (0.21)			0.11-0.30									
	2018 (n= 7791)		3233 (41.50)		40.40-42.59		645 (8.28)		7.67-8.89		23 (0.30)			0.17-0.42									

### Prevalence of Four Psychopathologies and Their Associations With Internet Addiction Severity

**Figure 1:**
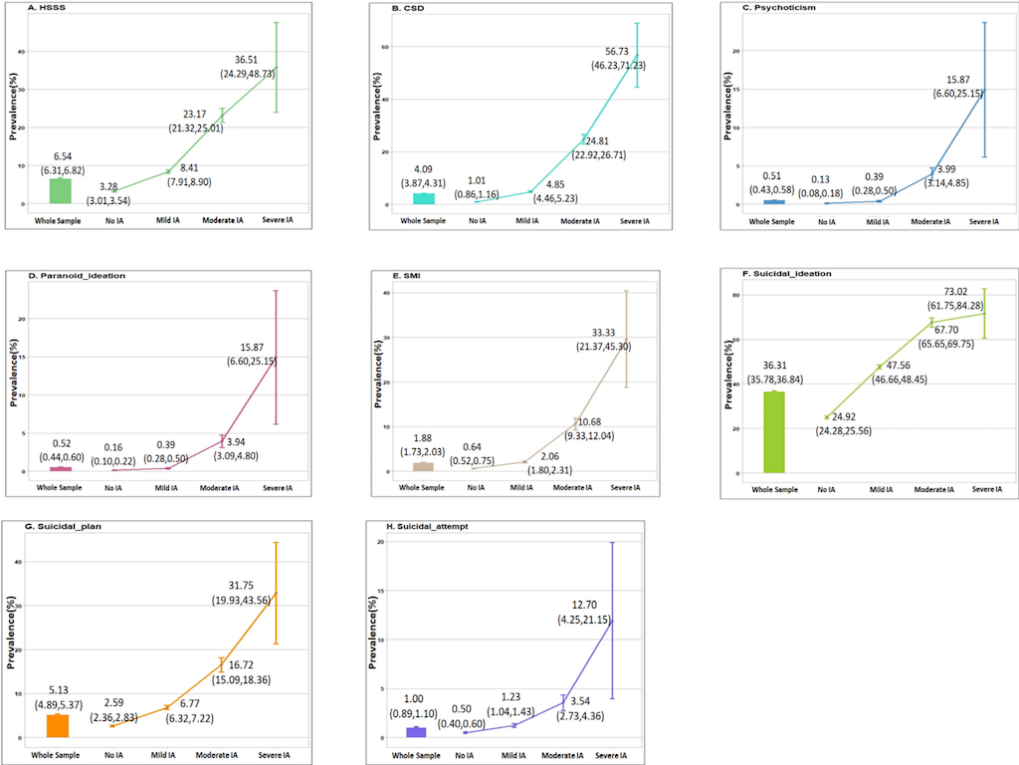
Prevalence rates (95% CI) of high somatic symptom severity (HSSS), clinically significant depression (CSD), psychoticism, paranoid ideation, serious mental illness (SMI), suicidal ideation, suicidal plan, and suicidal attempt in the whole sample, and in those without internet addiction (IA) and with mild, moderate, and severe IA.

Overall, the prevalence rates of high somatic symptom severity, depression, psychoticism, paranoid ideation, and serious mental illness in the current sample were 6.5%, 4.1%, 0.5%, 0.5%, and 1.9%; lifetime prevalence rates of suicidal ideation, suicidal plan, and suicidal attempt were 36.3%, 5.1%, and 1.0%, respectively. All of these rates significantly increased from the group without internet addiction to the groups with mild, moderate, and severe internet addiction. Compared with the average levels of the surveyed population, these rates in the group without internet addiction were much lower, whereas those in the group with mild internet addiction were similar (psychoticism, paranoid ideation, serious mental illness, and suicidal attempt) or mildly higher (high somatic symptom severity, clinically significant depression, suicidal ideation, and suicidal plan); however, these rates sharply increased in the moderate and severe internet addiction groups (Figure 1). Accordingly, the OR of each psychopathology and serious mental illness adjusted for demographics also significantly increased with internet addiction severity. ORs for each psychopathology in the moderate and severe internet addiction groups (except for that of clinically significant depression, which was most strongly associated with internet addiction) became significantly smaller when further adjusted for other psychopathologies (Table 2). In addition, although the adjusted ORs of suicidality also decreased after further adjusting for the four psychopathologies, they still increased significantly with internet addiction severity, especially for suicidal plans and suicidal attempts (Table 2). These associations were all in the same direction when analyzed for biological sex, although there were differences in the respective ORs (Multimedia Appendix 1 and Multimedia Appendix 2).

**Table 2:** Adjusted odds ratios (aORs) of mental health outcomes in the groups with mild, moderate, and severe internet addiction (IA).

Mental health outcome		Model 1^a^			Model 2^b^				
		aOR (95% CI)		*P* value			aOR (95% CI)		*P* value
**HSSS^c^**									
	Mild IA	2.61 (2.35-2.90)		<.001			2.30 (2.06-2.56)		<.001
	Moderate IA	8.24 (7.20-9.43)		<.001			4.32 (3.71-5.04)		<.001
	Severe IA	15.74 (9.27-26.73)		<.001			3.55 (1.90-6.64)		<.001
**CSD^d^**									
	Mild IA	4.86 (4.10-5.76)		<.001			4.17 (3.50-4.96)		<.001
	Moderate IA	30.55 (25.51-36.59)		<.001			18.88 (15.59-22.86)		<.001
	Severe IA	127.95 (75.57-216.64)		<.001			65.25 (35.72-119.20)		<.001
**Psychoticism**									
	Mild IA	2.91 (1.76-4.79)		<.001			1.92 (1.11-3.36)		.02
	Moderate IA	29.41 (18.40-47.00)		<.001			4.73 (2.63-8.50)		<.001
	Severe IA	128.71. (58.11-285.08)		<.001			7.12 (2.18-23.30)		.001
**Paranoid ideation**									
	Mild IA	2.29 (1.44-3.65)		<.001			1.46 (0.88-2.42)		.15
	Moderate IA	22.71 (14.76-34.96)		<.001			3.39 (1.94-5.95)		<.001
	Severe IA	101.76 (47.01-220.28)		<.001			5.16 (1.56-17.11)		.01
**SMI^e^**									
	Mild IA	3.14 (2.51-3.93)		<.001			N/A^f^		N/A
	Moderate IA	16.89 (13.34-21.37)		<.001			N/A		N/A
	Severe IA	67.97 (38.77-119.16)		<.001			N/A		N/A
**Suicidal ideation**									
	Mild IA	2.69 (2.56-2.83)		<.001			2.51 (2.39-2.64)		<.001
	Moderate IA	6.03 (5.45-6.66)		<.001			4.24 (3.81-4.71)		<.001
	Severe IA	7.75 (4.43-13.56)		<.001			3.07 (1.66-5.65)		<.001
**Suicidal plan**									
	Mild IA	2.60 (2.31-2.93)		<.001			2.24 (1.99-2.53)		<.001
	Moderate IA	6.77 (5.82-7.87)		<.001			3.31 (2.78-3.93)		<.001
	Severe IA	15.73 (9.12-27.14)		<.001			3.79 (2.03-7.09)		<.001
**Suicidal attempt**									
	Mild IA	2.35 (1.80-3.06)		<.001			1.98 (1.51-2.60)		<.001
	Moderate IA	6.41 (4.66-8.82)		<.001			2.60 (1.79-3.78)		<.001
	Severe IA	25.38 (11.66-55.22)		<.001			4.97 (2.07-11.94)		<.001

^a^Binary logistic regression adjusted for age, gender, and year of survey; no internet addiction group as reference (1).

^b^Binary logistic regression adjusted for ag, gender, year of survey, and psychopathologies; no internet addiction group as reference (1).

^e^HSSS: high somatic symptom severity, defined according to the total score of the Patient Health Questionnaire-15 using a cutoff of ≥10.

^d^CSD: clinically significant depression, defined according to the total score of the Patient Health Questionnaire-9 using a cutoff of ≥10.

^e^SMI: serious mental illness, defined according to the total score of the 6-item Kessler psychological distress scale using a cutoff of ≥13.

^f^N/A: not applicable.

### Rates of Comorbidity with the Four Psychopathologies and Their Associations with Internet Addiction Severity

Among all respondents, the rates of those who had at least one, two, and three of the four psychopathologies were 9.2%, 1.9%, and 0.4%, respectively. These rates increased significantly from the group without internet addiction to those with mild, moderate, and severe internet addiction (Table 3). Accordingly, the ORs based on the number of comorbid conditions adjusted for demographics also significantly increased with internet addiction severity (Table 3).

**Table 3:** Adjusted odds ratios (aORs) of the prevalence (%) of comorbidities of four psychopathologies^a^ in the groups with mild, moderate, and severe internet addiction (IA).

Number of comorbidities		Prevalence (95%CI)	aOR^b^ (95%CI)	*P* value
≥**1**				
	No IA	(3.76-4.35)	1	
	Mild IA	11.73 (11.15-12.30)	3.05 (2.78-3.35)	<.001
	Moderate IA	36.89 (34.78-39.01)	13.04 (11.57-14.69)	<.001
	Severe IA	68.25 (56.44-80.07)	48.00 (27.89-82.62)	<.001
≥**2**				
	No IA	0.41 (0.32-0.50)	1	
	Mild IA	2.02 (1.76-2.27)	4.84 (3.72-6.31)	<.001
	Moderate IA	13.98 (12.46-15.50)	36.68 (28.14-47.82)	<.001
	Severe IA	36.51 (24.29-48.73)	127.94 (72.51-225.73)	<.001
≥**3**				
	No IA	0.09 (0.04-0.13)	1	
	Mild IA	0.26 (0.17-0.35)	2.96 (1.60-5.49)	.001
	Moderate IA	3.74 (2.91-4.58)	42.84 (24.48-74.96)	<.001
	Severe IA	15.87 (6.60-25.15)	202.08 (86.50-472.09)	<.001

^a^The four psychopathologies are: high somatic symptom severity, clinically significant depression, psychoticism, and paranoid ideation.

^b^aOR: Adjusted odds ratio based on binary logistic regression analysis controlling for age, gender, and year of survey groups.

## Discussion

### Principal Findings and Research Priorities

The IAT, which is the most widely used internet addiction survey instrument in the world, allows researchers to differentiate the severity of internet addiction, as in the present study [10]. However, and likely a consequence of relatively small sample sizes, most previous studies used only the IAT to identify PIU using a total score cutoff at ≥50, which includes moderate to severe internet addiction; this issue clearly applies to prior research on the association of internet addiction with other mental health outcomes. The present study, which to our knowledge included the largest ever sample size in internet addiction research to date, is the first to use a methodology that enabled a comparison of prevalence rates and ORs of a series of representative psychopathologies, serious mental illness, and suicidality among groups without internet addiction, and with mild, moderate, and severe internet addiction. Consequently, this study has three prominent strengths in comparison with previous studies on associations between internet addiction and other mental health outcomes.

First, by comparing the prevalence rates and ORs of mental health outcomes among the differentiated internet addiction severity groups, the present study found a surprising incremental pattern of these conditions from the group without internet addiction through the group with severe internet addiction. That is, the prevalence rates of the four psychopathologies, serious mental illness, and suicidality in the group without internet addiction were much lower than the average levels of the surveyed population, and those in the group with mild internet addiction were similar to (psychoticism, paranoid ideation, serious mental illness, and suicidal attempt) or mildly higher than (high somatic symptom severity, clinically significant depression, suicidal ideation, and suicidal plan) the average levels of the surveyed population; however, the prevalence rates and their OR increments from mild to moderate and severe internet addiction were surprisingly large for most conditions. Previous surveys typically compared prevalence rates and ORs of individual syndromes between the groups without and with internet addiction. Based on this approach in the original studies, Ho et al [25] performed a large meta-analysis including eight studies comprising 1641 internet addiction cases and 11,210 controls, and the authors concluded that the relative ORs of ADHD symptoms, alcohol abuse, anxiety, and depression in internet addiction/PIU cases compared to those without internet addiction were respectively 3.05 (95% CI 2.14-4.37), 2.85 (95% CI 2.15-3.77), 2.77 (95% CI 2.04-3.75), and 2.70 (95% CI 1.46-4.97) [25]. However, due to the limited sample sizes in the studies included in the meta-analysis, there may not have been a sufficiently large number of severe internet addiction cases included in the analysis. Consequently, these studies failed to reveal either the dramatically increasing pattern of other mental health outcomes associated with internet addiction severity or the surprisingly high prevalence rates of other mental health outcomes in those with severe internet addiction. The prevalence rate of severe internet addiction might be lower, as noted in the present study, but is of paramount importance for an understanding of mental health outcomes associated with severe internet addiction, because most of the adverse events with a social impact related to internet exposure appear to be related mainly to such severe cases. By surveying the rates of a group of mental health outcomes in the mild internet addiction group, the present study also provides the first evidence that the rate of mental health problems is similar to or only mildly higher than the average level of the surveyed population and does not support the validity of mild internet addiction as a mental disorder.

Second, by investigating the largest number (four) of representative psychopathologies in the context of an internet addiction survey to date, the present study enabled an analysis of the overlap between these psychopathologies and their comorbidity, demonstrating a clear relationship with internet addiction severity. Previous surveys have typically reported an association of internet addiction with individual psychopathology because of a focus on the investigation of a single or very small number of psychopathologies. This may explain a possible significant underestimation of the prevalence of psychopathologies in groups with varying internet addiction severities, in addition to a lack of prior understanding of the pattern of overlap and comorbidity of the mental health outcomes linked to internet addiction. For example, the meta-analysis by Ho et al [25] showed that the prevalence of ADHD, alcohol abuse, anxiety, and depression among internet addiction/PIU cases was 21.7%, 13.3%, 23.3%, and 26.3%, respectively. However, the authors failed to report the rates and ORs of having at least one of these psychopathologies or having a comorbidity with multiple psychopathologies. Similar to the present study, the highest rate of common psychopathology among those with internet addiction/PIU in the meta-analysis was 26.3% for depression [25], but this rate was much lower than that of individuals with at least one of the four psychopathologies found in the present study (approximately 2/5 in the respondents with moderate internet addiction and more than 2/3 in those with severe internet addiction). Our approach also allowed us to observe the variability in the pattern of associations among the four psychopathology types. For example, clinically significant depression was less prevalent than high somatic symptom severity in the whole population, although the rate of clinically significant depression in the groups with moderate to severe internet addiction was the highest among the four psychopathologies. The rates of psychoticism and paranoid ideation were very low in the overall population but were substantial in the severe internet addiction cases and resulted in an approximately 100-fold increase in OR relative to the group without internet addiction after adjusting for demographics. When investigating the independent associations of internet addiction with other mental health outcomes by controlling for confounding effects of demographics and the interactions among psychopathologies, the present study provides empirical evidence for the previous presumption that depression might be the most strongly internet addiction–associated psychopathology [25], although other psychopathologies also remain independently associated with internet addiction. Another important finding from the present analyses that considered the confounding effect with psychopathologies was that the types of suicidality were also independently associated with internet addiction even after adjusting for both demographics and the presence of psychopathology. Cheng et al [26] documented that internet addiction (compared with no internet addiction) was independently associated with suicidal ideation and attempts in a meta-analysis, but they had not compared the associations among groups with distinct levels of internet addiction severity and had not compared these associations after controlling for the presence of psychopathology apart from depression.

Third, the present study is, to our knowledge, the first to investigate the association of internet addiction with high somatic symptom severity or serious mental illness. Consequently, it was found that internet addiction was strongly associated not only with mental symptoms, serious mental illness, and suicidality but also with somatic symptoms, which are the core features of many medical illnesses [39]. Given that serious mental illness screened by the K6 scale may include most DSM-IV/CIDI-10 mental disorders that result in serious impairment [37], the strong association of internet addiction with serious mental illness found in the present study is an important addition to previous research findings on the association of internet addiction with individual psychopathologies.

The overall prevalence rates of internet addiction (6.5%, including moderate and severe cases), psychopathologies (eg, 6.5% for high somatic symptom severity, 4.1% for clinically significant depression), and serious mental illness (1.9%) in the present sample of university freshmen were lower than those reported in other recent surveys (eg, 10.4% for PIU in Anhui, China [40]; 9.3% for high somatic symptom severity and 4.2% for clinically significant depression in general community populations reported by Kocalevent et al [31] and Yu et al [33], respectively; and 4.0% for serious mental illness in the whole undergraduate population at the same university [37]). The reasons for these differences may be complex but may be at least partly explained as follows. On the one hand, Asian people are less willing to express their mental health problems than their Western counterparts, and it has been reported that Chinese people tend to provide lower scores on mental health screening questionnaires than other races/ethnicities [36]. On the other hand, it has been reported that healthy people, physically or psychologically, might achieve more success at school, and they are expected to be more likely to enter national comprehensive universities [36]. The choice of a Sichuan University sample is likely relevant, as the present student sample may have collectively invested more time in preparation for the National College Entrance Examination, resulting in their high scores and matriculating into a leading-level Chinese university, which might be an indication that they had less casual internet time. However, the lifetime prevalence rates for suicidalities were not consistently lower than those of previous surveys. For example, the rate of suicidal ideation (36.3%) was similar to that found in a Polish adolescent group (31%) but was much higher than that reported for US adolescents (12.1%). The rate of suicidal plans (5.1%) was slightly higher than those in US adolescents (4.0%) and in a Korean adult population (3.3%). The rate of suicidal attempts (1.0%) was also slightly higher than that in a US adult population (0.8%), although it was much lower than that in US adolescents (4.1%) [41-43]. On a technical note, the reasons for the lower rate of suicidal attempts might partly be attributed to a translation problem relevant to cultural differences between Chinese and English-speaking people; although this issue is noteworthy, it has not been studied in previous research. That is, “attempted to kill myself” has usually been translated into “尝试自杀” (ChangShi ZiSha) in Chinese, which means that the “killing” behavior had already been conducted although it had not resulted in death; in contrast, the phrase in English may mean that the behavior of “killing” had not actually occurred.

Most previous studies have documented that either men are more vulnerable to internet addiction than women or that internet addiction is not gender-specific [36,37]. This is inconsistent with the present study, which found that the rates of moderate or severe internet addiction in women (7.2%) were higher than those in men (5.9%). This may be partially due to the increasing availability of more online games with gender equity or a female orientation (such as The Honor of Kings, Onmyoji) in recent years. A recent survey in the United Kingdom also reported that girls were more likely to be bothered by the content on the internet, which may be attributed to the fact that girls are more concerned about this problem than their male counterparts [44]. As the results have been inconsistent, it is too early to make conclusions regarding gender differences related to internet addiction. This study showed that the prevalence of internet addiction among freshmen increased in later years. This might be partly due to the increasing availability and convenience of the internet with the popularity of mobile phones and other mobile internet devices, and might indicate that increasing exposure to the internet environment may lead to a high risk of internet addiction.

### Limitations

The present study has several limitations. As a cross-sectional survey, it does not help to inform us of a possible causal relationship between internet addiction and comorbidity with other mental health outcomes. The use of self-report scales, as in most previous studies, is another obstacle to the generation of a clinical diagnosis. It is possible that comparative data for this student population on several annual diagnoses of mental disorders may be useful for supporting our use of survey self-report data in contrast to standard clinical diagnoses, but there are two issues arising in the current context. On the one hand, the number of annual diagnoses of mental disorders at the university clinic is not available for research analysis due to institutional data access policies related to personal information and protection of privacy for individual students. On the other hand, we expect that such data (if available) would be only partially helpful in view of an expected limitation of correspondence of the data of this study and the university clinic data. Disparities between prevalence rates based on mental health clinic presentation–based estimates and community survey–based estimates are expected. Epidemiological studies have clearly documented that health care–seeking rates of individuals with mental disorders in general communities is low, especially in low and middle income countries such as China, which provides partial support for our current survey approach rather than a medical records–based analysis. It has been argued that cultural issues leading to increased stigmatization and alternate support–seeking behavior may underly this pattern of reporting [45,46].

This study investigated internet addiction but we were not able to identify IGD (which is the focus of the most recent revisions of international disease classification and diagnostic instruments) nor did we identify the proportion of the student sample using the internet predominantly to access online games as opposed to other content such as social media and information. Further information on the proportion of different internet activities that participants engaged in will be necessary to achieve any detailed understanding of the relationship of internet use with addiction and other psychopathologies that are a significant concern for the healthy development of our youth in the information age. To gain insight into the etiology and possible therapeutic interventions for internet addiction and IGD, further research must include data on the proportional access of participants to online gaming, social media, and social messaging as well as access to other risk-related content such as pornography.

We investigated a broader group of mental health outcomes as possibly being associated with internet addiction than previous studies, but did not include all mental health factors. Our participants were first-year students at a single university, although they were from all of the provincial administrative regions of China. The gross enrollment rate in higher education in China from 2015 to 2018 ranged from 40% to 48.1% [47]. Healthy people, physically or psychologically, with better socioecological status typically have higher school achievements, and are more likely to enter national comprehensive universities [48,49]. Given this demographic profile of our sample, we must acknowledge that our findings may not apply generally to the overall Chinese population for this age range. Although the sample size of this study is very large, the small proportion of participants with severe internet addiction (n=63) hindered further analyses to control for the confounding effects of more detailed covariates such as home provinces (n>30) of participants. Future research with larger representative sample sizes and those adopting a longitudinal design approach will be advantageous. The inclusion of a structured clinical diagnostic interview for detailed internet addiction patterns and mental disorders, along with measurement of other relevant markers (such as genetic, neuroimaging, and treatment response) will further shed light on how to treat internet addiction from a perspective of effectively improving human health in the era of “Internet Plus” and artificial intelligence.

### Conclusions

The present study showed that internet addiction is strongly associated with a broad group of mental health problems (among which clinically significant depression was the most strongly associated), which strongly supports the illness validity of moderate and severe, but not mild, internet addiction. This study is also meaningful for health policy makers and service suppliers to manage the so-called internet addiction or other mental health outcomes relevant to internet usage, especially from the perspective of resolving the overall human health burden in the current era of “Internet Plus” and artificial intelligence.

## Appendix

Multimedia Appendix 1 Adjusted odds ratios (ORs) of the four psychopathologies and serious mental illness (SMI) of male and female respondents in the groups with mild, moderate, and severe internet addiction (IA).

Multimedia Appendix 2 Adjusted odds ratios (ORs) of suicidal ideation, suicidal plan, and suicidal attempt in the groups with mild, moderate, and severe internet addiction (IA) among males and females.

## Abbreviations

ADHD: attention deficit and hyperactivity disorder.
CIDI: Composite International Diagnostic Interview
DSM: Diagnostic and Statistical Manual of Mental Disorders
IAT: Young 20-item Internet Addiction Test
ICD: International Classification of Diseases
IGD: internet gaming disorder
K6: six-item Kessler psychological distress scale
OPHS: Online Psychosomatic Health Survey
OR: odds ratio
PHQ: Patient Health Questionnaire
PIU: problematic internet use
SBQ-R: Suicidal Behaviors Questionnaire-Revised
SCL-90: Symptom Checklist 90
