# Supplementary material for: Associations of Internet Addiction Severity With Psychopathology, Serious Mental Illness, and Suicidality: Large-Sample Cross-Sectional Study
Source: J Med Internet Res. 2020 Aug 11;22(8):e17560. doi: 10.2196/17560 (PMC7448182; doi:10.2196/17560)
Supplement: Multimedia Appendix 1 [file jmir_v22i8e17560_app1.docx]

**Multimedia Appendix 1. The adjusted ORs of the four psychopathologies and SMI of males and females respondents in the groups with mild, moderate and severe IA^a^.**

|  | **Males** | | | |  | **Females** | | | |
| --- | --- | --- | --- | --- | --- | --- | --- | --- | --- |
|  | **aOR^b^ (95% CI)^c^** | ***p*** | **aOR^d^ (95% CI)^c^** | ***p*** |  | **aOR^b^ (95% CI)^c^** | ***p*** | **aOR^d^ (95% CI)^c^** | ***p*** |
| **HSSS^e^** |  |  |  |  |  |  |  |  |  |
| No IA | 1 |  | 1 |  |  | 1 |  | 1 |  |
| Mild IA | 2.90 (2.40, 3.49) | <.001 | 2.53 (2.09, 3.06) | <.001 |  | 2.48 (2.18, 2.82) | <.001 | 2.19 (1.92, 2.49) | <.001 |
| Moderate IA | 10.87 (8.67, 13.62) | <.001 | 5.73 (4.45, 7.39) | <.001 |  | 7.06 (5.97, 8.36) | <.001 | 3.68 (3.04, 4.46) | <.001 |
| Severe IA | 18.01 (7.85, 41.32) | <.001 | 4.45 (1.66, 11.93) | 0.003 |  | 14.80 (7.44, 29.44) | <.001 | 3.13 (1.40, 6.98) | 0.005 |
| **CSD^f^** |  |  |  |  |  |  |  |  |  |
| No IA | 1 |  | 1 |  |  | 1 |  | 1 |  |
| Mild IA | 5.06 (3.89, 6.58) | <.001 | 4.40 (3.36, 5.76) | <.001 |  | 4.73 (3.78, 5.91) | <.001 | 4.02 (3.20, 5.05) | <.001 |
| Moderate IA | 30.98 (23.42, 41.00) | <.001 | 18.74 (13.89, 25.27) | <.001 |  | 30.20 (23.86, 38.24) | <.001 | 18.88 (14.71, 24.22) | <.001 |
| Severe IA | 104.03 (48.32, 223.99) | <.001 | 56.75 (23.29, 138.27) | <.001 |  | 156.18 (74.08, 329.23) | <.001 | 74.60 (32.29, 172.34) | <.001 |
| **Psychoticism** |  |  |  |  |  |  |  |  |  |
| No IA | 1 |  | 1 |  |  | 1 |  | 1 |  |
| Mild IA | 3.04 (1.56, 5.93) | 0.001 | 1.57 (0.73, 3.40) | 0.25 |  | 2.71 (1.27, 5.76) | 0.01 | 2.18 (0.96, 4.97) | 0.06 |
| Moderate IA | 30.04 (15.94, 56.61) | <.001 | 4.59 (2.01, 10.47) | <.001 |  | 28.20 (14.02, 56.71) | <.001 | 4.31 (1.84, 10.10) | 0.001 |
| Severe IA | 101.77 (30.83, 335.98) | <.001 | 6.34 (0.98, 41.11) | 0.05 |  | 160.34 (54.05, 475.61) | <.001 | 8.75 (1.89, 40.59) | <.001 |
| **Paranoid ideation** | |  |  |  |  |  |  |  |  |
| No IA | 1 |  | 1 |  |  | 1 |  | 1 |  |
| Mild IA | 2.82 (1.50, 5.28) | 0.001 | 1.98 (1.00, 3.93) | 0.049 |  | 1.81 (0.90, 3.61) | 0.09 | 1.05 (0.49, 2.25) | 0.91 |
| Moderate IA | 18.63 (9.93, 34.95) | <.001 | 2.86 (1.25, 6.54) | 0.01 |  | 26.28 (14.41, 47.95) | <.001 | 3.62 (1.65, 8.01) | 0.001 |
| Severe IA | 60.17 (16.30, 222.06) | <.001 | 2.54 (0.35, 18.39) | 0.36 |  | 144.34 (53.71, 387.87) | <.001 | 6.76 (1.49, 30.69) | 0.01 |
| **SMI^g^** |  |  |  |  |  |  |  |  |  |
| No IA | 1 |  | - |  |  | 1 |  | - |  |
| Mild IA | 3.13 (2.20, 4.44) | <.001 | - |  |  | 3.13 (2.33, 4.20) | <.001 | - |  |
| Moderate IA | 17.26 (11.92, 25.00) | <.001 | - |  |  | 16.59 (12.22, 22.52) | <.001 | - |  |
| Severe IA | 63.91 (26.77, 152.59) | <.001 | - |  |  | 71.94 (34.36, 150.64) | <.001 | - |  |

^a^IA: internet addiction.

^b^aOR: adjusted odds ratio based on binary logistic regression analysis, controlling for age category and the year-of-survey groups.

^c^95% CI, 95% confidence interval.

^d^aOR: adjusted odds ratio based on binary logistic regression analysis, controlling for age category, the year-of-survey groups and psychopathologies.

^e^HSSS: high somatic symptom severity, which was defined according to the total score of the Patient Health Questionnaire-15 (PHQ-15) using a cutoff ≥ 10.

^f^CSD: clinically significant depression, which was defined according to the total score of the Patient Health Questionnaire-9 (PHQ-9) using a cutoff ≥ 10.

^g^SMI: severe mental illness, which was defined according to the total score of the 6-item Kessler psychological distress scale (K6) using a cutoff ≥ 13.
