# Supplementary material for: Associations of Internet Addiction Severity With Psychopathology, Serious Mental Illness, and Suicidality: Large-Sample Cross-Sectional Study
Source: J Med Internet Res. 2020 Aug 11;22(8):e17560. doi: 10.2196/17560 (PMC7448182; doi:10.2196/17560)
Supplement: Multimedia Appendix 2 [file jmir_v22i8e17560_app2.docx]

**Multimedia Appendix 2. The adjusted ORs of suicidal ideation, suicidal plan and suicidal attempt in the groups with mild, moderate and severe IA^a^ among males and females.**

|  | **Males** | | | |  | **Females** | | | |
| --- | --- | --- | --- | --- | --- | --- | --- | --- | --- |
|  | **aOR^b^ (95% CI)^c^** | ***p*** | **aOR^d^ (95% CI)^c^** | ***p*** |  | **aOR^b^ (95% CI)^c^** | ***p*** | **aOR^d^ (95% CI)^c^** | ***p*** |
| **Suicidal ideation** |  |  |  |  |  |  |  |  |  |
| No IA | 1 |  | 1 |  |  | 1 |  | 1 |  |
| Mild IA | 2.62 (2.44,2.81) | <.001 | 2.48 (2.31, 2.66) | <.001 |  | 2.75 (2.56, 2.95) | <.001 | 2.54 (2.36, 2.72) | <.001 |
| Moderate IA | 5.26 (4.57, 6.06) | <.001 | 3.89 (3.35, 4.51) | <.001 |  | 6.85 (5.94, 7.90) | <.001 | 4.62 (3.97, 5.37) | <.001 |
| Severe IA | 5.29 (2.49, 11.23) | <.001 | 2.45 (1.07, 5.61) | 0.03 |  | 12.12 (5.00, 29.38) | <.001 | 4.00 (1.55, 10.34) | 0.004 |
| **Suicidal plan** |  |  |  |  |  |  |  |  |  |
| No IA | 1 |  | 1 |  |  | 1 |  | 1 |  |
| Mild IA | 2.57 (2.14, 3.08) | <.001 | 2.22 (1.84, 2.67) | <.001 |  | 2.63 (2.26, 3.07) | <.001 | 2.27 (1.94, 2.66) | <.001 |
| Moderate IA | 5.58 (4.35, 7.15) | <.001 | 2.59 (1.94, 3.46) | <.001 |  | 7.60 (6.27, 9.21) | <.001 | 3.82 (3.07, 4.75) | <.001 |
| Severe IA | 10.44 (4.18, 26.08) | <.001 | 2.36 (0.82, 6.81) | 0.11 |  | 20.62 (10.23, 41.58) | <.001 | 5.00 (2.26, 11.04) | <.001 |
| **Suicidal attempt** |  |  |  |  |  |  |  |  |  |
| No IA | 1 |  | 1 |  |  | 1 |  | 1 |  |
| Mild IA | 2.35 (1.52, 3.65) | <.001 | 2.01 (1.29, 3.15) | 0.002 |  | 2.33 (1.67, 3.26) | <.001 | 1.95 (1.39, 2.74) | <.001 |
| Moderate IA | 6.19 (3.56, 10.76) | <.001 | 2.53 (1.33, 4.84) | 0.005 |  | 6.49 (4.38, 9.60) | <.001 | 2.59 (1.64, 4.10) | <.001 |
| Severe IA | 28.22 (8.10, 98.36) | <.001 | 5.70 (1.37, 23.67) | 0.02 |  | 24.08 (8.92, 65.00) | <.001 | 4.75 (1.58, 14.31) | 0.006 |

^a^IA: internet addiction.

^b^aOR: adjusted odds ratio based on binary logistic regression analysis, controlling for age category and the year-of-survey groups.

^c^95% CI, 95% confidence interval.

^d^aOR: adjusted odds ratio based on binary logistic regression analysis, controlling for age category, the year-of-survey groups and four psychopathologies (HSSS, CSD, psychoticism and paranoid ideation).
